# Categorical versus dimensional structure of autism spectrum disorder: A multi‐method investigation

**DOI:** 10.1002/jcv2.12142

**Published:** 2023-02-21

**Authors:** Thomas W. Frazier, Lacey Chetcuti, Fouad A. Al‐Shaban, Nick Haslam, Iman Ghazal, Eric W. Klingemier, Mohammed Aldosari, Andrew J. O. Whitehouse, Eric A. Youngstrom, Antonio Y. Hardan, Mirko Uljarević

**Affiliations:** ^1^ Department of Psychology John Carroll University University Heights Ohio USA; ^2^ Olga Tennison Autism Research Centre School of Psychology and Public Health La Trobe University Melbourne Victoria Australia; ^3^ Neurological Disorders Research Center Qatar Biomedical Research Institute Hamad Bin Khalifa University Doha Qatar; ^4^ Melbourne School of Psychological Sciences University of Melbourne Melbourne Victoria Australia; ^5^ Caregiver Experience Cleveland Clinic Cleveland Ohio USA; ^6^ Neurological Institute Cleveland Clinic Cleveland Ohio USA; ^7^ Telethon Kids Institute University of Western Australia Perth Western Australia Australia; ^8^ Department of Psychology and Neuroscience University of North Carolina at Chapel Hill Chapel Hill North Carolina USA; ^9^ Department of Psychiatry and Behavioral Sciences Stanford University Stanford California USA

**Keywords:** autism, categorical, dimensional, latent, taxometric

## Abstract

**Background:**

A key question for any psychopathological diagnosis is whether the condition is continuous or discontinuous with typical variation. The primary objective of this study was to use a multi‐method approach to examine the broad latent categorical versus dimensional structure of autism spectrum disorder (ASD).

**Method:**

Data were aggregated across seven independent samples of participants with ASD, other neurodevelopmental disorders (NDD), and non‐ASD/NDD controls (aggregate *N*s = 512–16,755; ages 1.5–22). Scores from four distinct phenotype measures formed composite “indicators” of the latent ASD construct. The primary indicator set included eye gaze metrics from seven distinct social stimulus paradigms. Logistic regressions were used to combine gaze metrics within/across paradigms, and derived predicted probabilities served as indicator values. Secondary indicator sets were constructed from clinical observation and parent‐report measures of ASD symptoms. Indicator sets were submitted to taxometric‐ and latent class analyses.

**Results:**

Across all indicator sets and analytic methods, there was strong support for categorical structure corresponding closely to ASD diagnosis. Consistent with notions of substantial phenotypic heterogeneity, the ASD category had a wide range of symptom severity. Despite the examination of a large sample with a wide range of IQs in both genders, males and children with lower IQ were over‐represented in the ASD category, similar to observations in diagnosed cases.

**Conclusions:**

Our findings provide strong support for categorical structure corresponding closely to ASD diagnosis. The present results bolster the use of well‐diagnosed and representative ASD groups within etiologic and clinical research, motivating the ongoing search for major drivers of the ASD phenotype. Despite the categorical structure of ASD, quantitative symptom measurements appear more useful for examining relationships with other factors.

## Graphical abstract

This study addressed the question of whether autism spectrum disorder (ASD) is best represented as a continuum (A) or a category (B). Results provided strong support for the categorical structure of ASD. Consistent with notions of substantial phenotypic heterogeneity, the ASD category had a wide range of symptom severity.

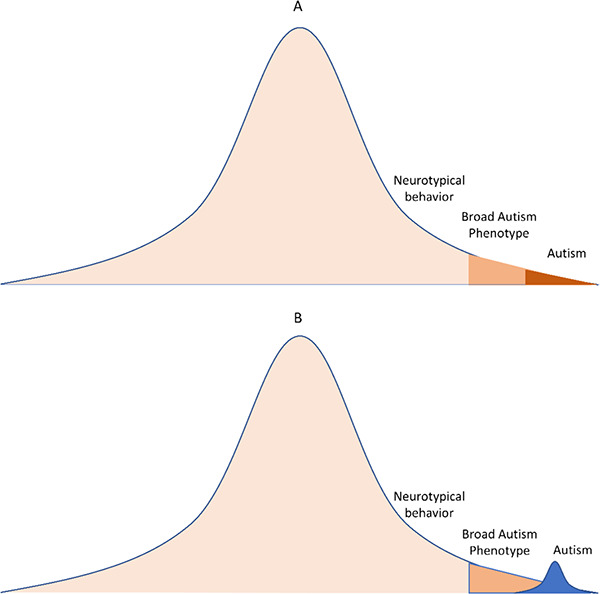

**Key points**

- Although several studies have addressed the question of whether autism spectrum disorder (ASD) is best represented as a category or continuum, results have been inconsistent.
- This was the first study to implement a multi‐method approach, using datasets an order of magnitude larger than prior analyses.
- Results were consistent across multiple different types of measures. The inclusion of non‐ASD neurodevelopmental disorder (NDD) and non‐ASD/NDD controls provided a strong test of categorical structure.
- Our findings provide strong support for the categorical structure of ASD, with the category corresponding closely to clinical diagnoses. Consistent with notions of substantial phenotypic heterogeneity, the ASD category had a wide range of symptom severity.

## INTRODUCTION

A fundamental question for any neuropsychiatric diagnosis concerns whether the condition is best represented using a categorical or dimensional framework. A recent meta‐analysis (Haslam et al., 2020) suggested that, although most psychiatric disorders and constructs are best represented as dimensional, autism spectrum disorder (ASD) is among several possible exceptions (Frazier et al., 2010, 2012; James et al., 2016). However, exclusive focus on subjective measures could have biased ASD findings toward categorical conclusions (Beauchaine & Waters, 2003; Ruscio, 2007). Therefore, there is a need for large sample, multi‐modal investigations spanning eye‐tracking, clinical observations, and questionnaires.

The question of categorical versus dimensional structure has substantial implications for conceptual models and assessment (Ruscio & Ruscio, 2002). Under a dimensional model, neurobiological research would emphasize quantitative structural and functional changes, while clinical assessment would focus on obtaining a precise symptom severity estimate and linking this estimate with relevant phenotypic features such as functional capacity (Ruscio & Ruscio, 2002). A categorical model would instead support the search for qualitatively distinct structural and functional imaging indicators and focus on optimizing instruments to generate a post‐test probability of ASD diagnosis (Frazier, Coury, et al., 2021).

Converging evidence suggests that individuals with ASD form a distinct latent subpopulation with social communication/interaction (SCI) and restricted/repetitive behavior (RRB) core features that are qualitatively different from the remainder of the population. For example, ASD diagnosis has high inter‐rater reliability (Regier et al., 2013) and temporal stability (Pierce et al., 2019) from early life. Moreover, SCI and RRB co‐occur more than would be expected by chance in subsets of cases with pathogenic mutations (Morris et al., 2016). Twin studies further support a strong genetic component to ASD (Sandin et al., 2017), yet with nonshared environmental factors responsible for variation in severity of symptomatology above the diagnostic threshold (Castelbaum et al., 2020). At the same time, several pieces of data support a dimensional model. For example, there is considerable heterogeneity of severity and expression within the ASD phenotype (Lord et al., 2020). There is also evidence of a subthreshold or broad autism phenotype (BAP) across the general population (Piven et al., 1997; Sucksmith et al., 2011) with twin research designs suggesting similar etiology between typical and extreme symptom levels (Lundstrom et al., 2012) and partially distinct etiology for symptom domains (Ronald et al., 2006). Furthermore, a small subset of ASD cases no longer meet diagnostic criteria as they progress through development (Fein et al., 2013). The present study aimed to shed light on these seemingly conflicting observations by utilizing datasets an order of magnitude larger than previous investigations that span ASD, non‐ASD neurodevelopmental disorder (NDD) and non‐ASD/NDD controls.

This is the first study to date to combine a multi‐measurement approach—spanning eye‐tracking, clinical observation scales, and informant‐reported ASD measures—and to include NDD and non‐ASD/NDD controls for a strong test of categorical structure. The inclusion of multi‐assessment modalities is crucial for ensuring an unbiased evaluation of latent structure. Subjective report measures can be biased toward categorical or dimensional structure, depending on whether they were designed as screening or quantitative assessments (Baron‐Cohen et al., 2001; Beauchaine & Waters, 2003; Constantino & Gruber, 2012), and clinical observation measures may be biased toward categorical structure because clinicians are often implicitly comparing to a diagnostic prototype (Beauchaine & Waters, 2003). Gaze measures are objective and a substantial body of evidence has found consistent differences in social attention between ASD and non‐ASD cases (Frazier et al., 2017), with powerful differentiation when combining gaze measures across multiple distinct stimuli (Frazier et al., 2018).

**FIGURE 1:**
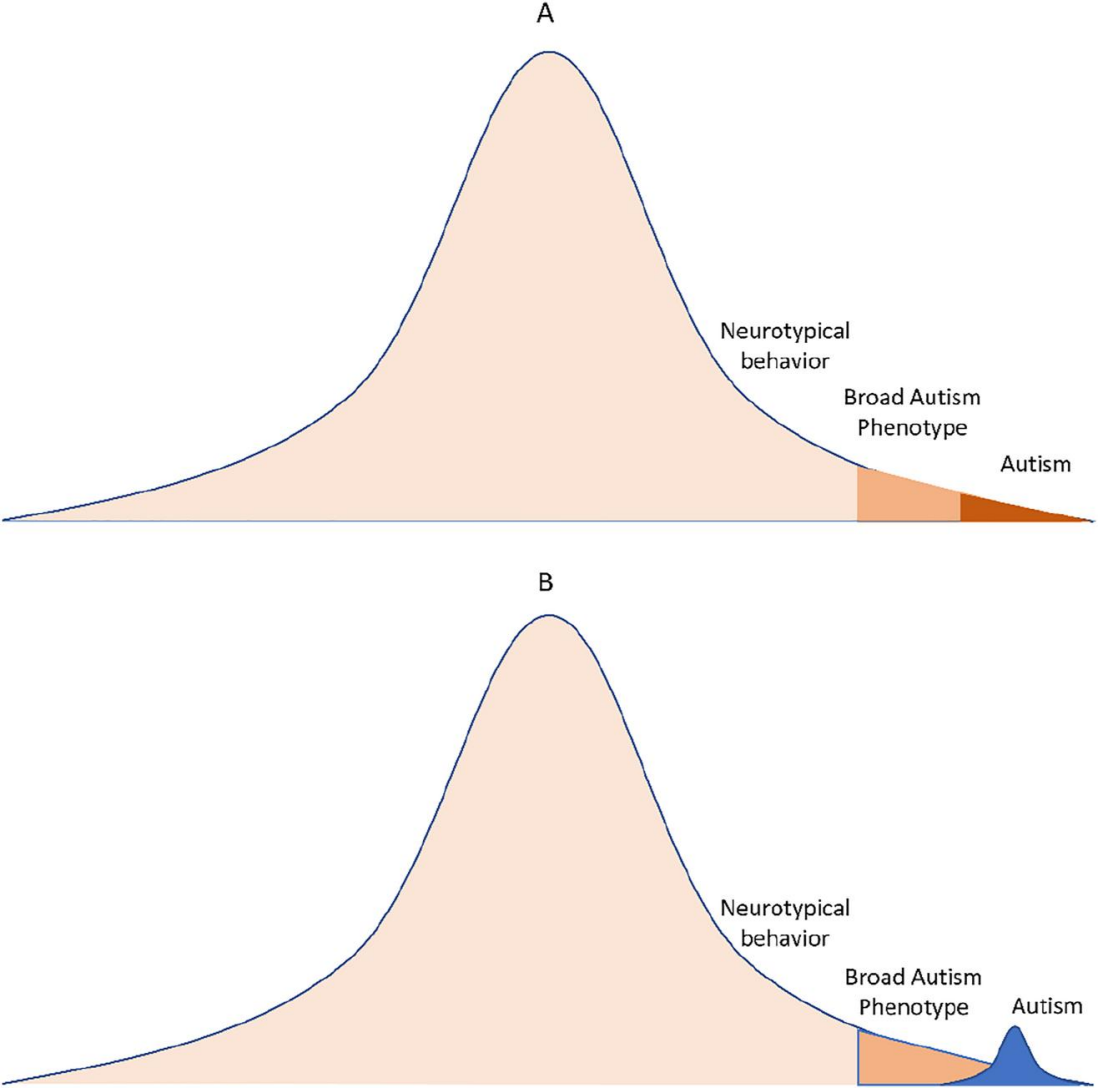
Dimensional (A) versus categorical (B) models of autism.

The primary aim of the present study was to examine whether ASD is best represented as a distinct category or as part of a continuum that includes neurotypical behavior (Figure 1). Based on previous evidence, ASD was expected to show categorical structure, with a wide severity range, across all samples and measures. While different measurement modalities were anticipated to converge on categorical structure, subjective reports might overestimate the ASD category base rate due to high sensitivity but low specificity (Moody et al., 2017), while observational measures might slightly underestimate the ASD category base rate due to moderate‐to‐good sensitivity and specificity (Hus & Lord, 2014). Should an ASD category be identified, the study aimed to compare the category base rate across statistical procedures and with clinical ASD diagnoses and characterize demographic and clinical correlates. Assignment to the putative ASD category identified from taxometric procedures was expected to correspond with high sensitivity and specificity to clinical ASD diagnosis, which have been shown to have good test‐retest and inter‐rater reliability (Lord, Petkova, et al., 2012; Regier et al., 2013). Latent category classifications were also expected to associate strongly with symptom severity measures, show substantial male bias, and yield significant associations with measures of IQ (Charman et al., 2011) and psychopathology due to diagnostic comorbidity (Hawks & Constantino, 2019; Simonoff et al., 2008).

## METHOD

### Participants

Indicator sets submitted to taxometric and latent structure analyses were derived from seven independent samples: Qatar Foundation and US‐Cleveland Clinic (Autism EYES; Frazier, Uljarevic, et al., 2021), Simons Simplex Collection (SSC; Fischbach & Lord, 2010), the Autism Genetic Resource Exchange (AGRE; Geschwind et al., 2001), National Database for Autism Research (NDAR; Hall et al., 2012), Social Responsiveness Scale normative data (SRS Norm; Constantino & Gruber, 2012), and Healthy Brain Network (HBN; Alexander et al., 2017). Each sample is characterized in Table S1. Samples were combined to create aggregate datasets. Combined samples included ASD and NDD clinical cases and non‐clinical (non‐ASD/NDD) controls, making it unlikely categorical structure would result simply from combining different samples.

### Diagnostic procedures

Each dataset included information on the presence/absence of ASD, other NDD diagnoses, or whether the participant was a non‐ASD/NDD control. ASD diagnoses were informant‐reported or based on best estimate clinical or research diagnoses, and informed by validated and gold‐standard assessment instruments including the Autism Diagnostic Interview‐Revised (ADI‐R; Lord et al., 1994) and/or Autism Diagnostic Observation Schedule (ADOS; Lord, Rutter, et al., 2012; Table S2). The NDD group comprised cases with any other neurodevelopmental condition besides ASD, ascertained from informant‐reported clinical diagnosis or clinical/research evaluation. Diagnostic data were coded to reflect three groups (ASD, NDD, non‐ASD/NDD) and, where applicable, the NDD and non‐ASD/NDD groups were combined to generate a single control group for comparison to ASD.

### Measures and indicator sets

Gaze data were obtained from the combined US and Qatar cohorts, which have previously been shown to have minimal differences and similar developmental patterns (Frazier, Uljarevic, et al., 2021). Fixation time percent, fixation count, and average fixation duration were recorded in response to 44 stimuli from seven paradigms (Frazier et al., 2018). The ADOS is a clinician‐observation measure of autism symptoms (Lord, Rutter, et al., 2012). The measure includes five modules (toddler and modules 1–4) that are administered dependent on age and speech/language status. Only data from modules 1–4 were included in the present study to maximize item overlap. The SRS is a parent‐report, 65‐item quantitative assessment of the severity of autism traits (Constantino & Gruber, 2012). The lifetime version of the Social Communication Questionnaire (SCQ) is a parent‐report dichotomously keyed (yes/no) rating scale that consists of 40 questions many of which tap DSM‐IV‐TR symptom domains (Rutter et al., 2003). Lifetime ratings reference the child's behavior throughout their developmental history, increasing diagnostic validity (Lord et al., 1997).

Four indicator sets were created from the gaze datasets. Logistic/linear regressions were used to predict ASD diagnosis from all available gaze metrics for each of the seven paradigms (Gaze‐7‐dx) and each gaze metric across the seven paradigms (Gaze‐3‐dx) and to predict quantitative ASD trait scores (derived from the SCQ and SRS) from the same sets of variables (Gaze‐7‐qt and Gaze‐3‐qt). The predicted values derived from these regressions served as indicator values, averaged together to derive indicator sets. This construction strategy ensured that the indicators/sets were sufficiently valid for taxometric analysis (Ruscio et al., 2006). In addition, we have also applied taxometric procedures to gaze metrics in their raw form.

Two indicator sets were constructed from the ADOS dataset. The first (ADOS‐Items) included eight items assessing core ASD symptoms that do not require speech and are common across modules 1–4 (eye contact, shared enjoyment, response to joint attention bids, imagination, quality of social overtures, gestures, unusual sensory interest, complex mannerisms). The second indicator set (ADOS‐Sums) reflected the average of three sum scores, derived from items common to modules 2–4: (i) social affect items assessing non‐verbal communication (gestures, eye contact, response to joint attention bids), (ii) items assessing reciprocal social behavior (non‐echoed speech, conversation, shared enjoyment, quality of social overtures, quality of social response, reciprocal social interaction, quality of rapport, imagination), and (iii) items assessing unusual repetitive and sensory behavior (speech abnormalities, immediate echolalia, stereotyped words, unusual sensory interests, and complex mannerisms). Table S3 lists ADOS items comprising each indicator set.

Three indicator sets were created from the SRS dataset. The first included all of the original SRS subscale scores (SRS‐Original; Constantino & Gruber, 2012). The second included subscales derived from a prior factor analysis of population data (SRS‐Factors; Frazier et al., 2014). The third consisted of subscales derived from recent analyses focused on mapping items to National Institute of Mental Health Research Domain Criteria (RDoC; Insel et al., 2010; Uljarevic et al., 2019) or identifying specific RRB groupings (SRS‐RDoC; Uljarević et al., 2021). Indicators in the SCQ set were based on recent factor analyses (Uljarevic et al., 2020; Uljarević et al., 2021).

Gaze‐7‐dx was considered the primary indicator set for the present study given high reliability and expected desirable properties (indicator validity, minimal skew, and low nuisance correlations). However, interpretation of taxometric results relied on convergence across indicator sets, samples, and procedures (Ruscio et al., 2006).

### Data analysis

Three taxometric procedures were implemented in R using default values from the RTaxometrics package (Ruscio & Wang, 2020): mean above minus below a cut (MAMBAC) (Meehl & Yonce, 1994) calculates the mean difference on one indicator set for cases falling above and below a sliding cut‐off score on another indicator set in search of an optimal cutting score to separate groups (should they exist); maximum eigenvalue (MAXEIG; Meehl & Yonce, 1996) organizes one input indicator set sequentially into overlapping windows and, at each window, calculates the first eigenvalue of a modified covariance matrix for all remaining indicators; latent mode (L‐Mode; Waller & Meehl, 1998) graphs the distribution of scores on the first principal factor of the full set of indicators. Each procedure was repeated using all possible indicator set combinations yielding individual and averaged graphical output, with categorical structure evidenced by peaked MAMBAC and MAXEIG curves and bi‐modal L‐mode distribution. Each procedure further provided an estimate of the base rate (or prevalence) of membership in the putative ASD category.

Comparison curves were generated for dimensional and categorical samples that reproduced the characteristics of the empirical data. Simulated curves were compared to empirical data curves using the comparison curve fit index (CCFI; Ruscio et al., 2018), derived from the root‐mean‐square residual estimates of each model. CCFI values discriminate dimensional and categorical structure with high accuracy under a wide range of data conditions (Ruscio et al., 2018). The CCFIs for each procedure were averaged to produce a mean CCFI. CCFI values < 0.50 support dimensional structure and >0.50 support categorical structure. Values between 0.45 and 0.55 were considered weak support, while values <0.45 and >0.55 were considered strong support for dimensional and categorical structure, respectively (Ruscio et al., 2018). Convergence of taxometric results across the different indicator sets and procedures further indicated robustness of the structural solution (Ruscio et al., 2006). Taxometric analyses were supplemented with latent class analyses (LCA), computed for each indicator set using maximum likelihood estimation with robust standard errors (see Supplemental Methods in Supporting Information S1). This permitted evaluation of whether LCA classifications overlap with diagnostic classifications (kappa, % accuracy, sensitivity, and specificity). Considering taxometric procedures are unable to detect the existence of more than two latent distributions, LCA further permitted evaluation of structures with up to five latent categories.

## RESULTS

### Indicator set characteristics

Combined samples for each indicator set had a diverse set of characteristics and were well‐above the recommended minimum size (*N* = 300; Table 1). Average indicator validity was highly variable, with lower than desired levels (*d* ≥ 1.25) for Gaze‐7‐dx, Gaze‐7‐qt, and SCQ. Average indicator skew was within the desired range (skew <1.0) for all indicator sets. Average nuisance correlations tended to be higher than the optimal upper bound (*r* < 0.30), especially for Gaze‐3‐dx, Gaze‐3‐qt, and SRS‐Original. These deviations from desired indicator set characteristics would be expected to decrease the likelihood of identifying categorical structure (Ruscio et al., 2010).

**TABLE 1:** Indicator set characteristics.

Set	# of indicators	Total	Non‐ASD/NDD	NDD	ASD	Validity (*d*)^a^	Skew	ASD *r* ^b^	Control *r* ^b^
		*N*	*N*	*N*	*N*	M (range)	M (range)	M	M
Gaze‐7‐dx	7	512	145	122	245	0.85 (0.58 to 1.28)	0.26 (−0.12 to 0.56)	0.36	0.27
Gaze‐3‐dx	3	512	145	122	245	1.29 (1.15 to 1.43)	0.23 (0.15 to 0.33)	0.71	0.60
Gaze‐7‐qt	7	512	145	122	245	0.57 (0.24 to 0.96)	0.48 (−0.01 to 1.67)	0.33	0.33
Gaze‐3‐qt	3	512	145	122	245	1.30 (1.16 to 1.44)	0.23 (0.15 to 0.33)	0.71	0.60
ADOS‐Items	8	12,705	864	1606	10,235	1.25 (0.99 to 2.11)	0.31 (−0.97 to 0.85)	0.28	0.14
ADOS‐Sums	3	8144	392	1255	6497	1.78 (1.63 to 1.98)	0.30 (−0.12 to 0.56)	0.41	0.26
SRS‐original	5	16,755	4961	1672	10,122	1.84 (1.55 to 2.01)	0.19 (0.04 to 0.38)	0.59	0.73
SRS‐factors	5	16,755	4961	1672	10,122	1.32 (0.24 to 1.91)	0.99 (−0.04 to 2.40)	0.32	0.39
SRS‐RDoC	7	16,755	4961	1672	10,122	1.31 (0.71 to 1.69)	0.63 (0.14 to 1.11)	0.30	0.50
SCQ	6	6040	1320	2004	2716	0.68 (0.58 to 1.01)	0.75 (0.28 to 0.99)	0.23	0.33

Abbreviations: ADOS, Autism Diagnostic Observation Schedule; ASD, autism spectrum disorder; dx, indicator set based on prediction of diagnosis; NDD, neurodevelopmental disorder (non‐ASD) controls; qt, indicator set based on prediction of quantitative trait/symptom measure; RDoC, Research Domain Criteria; SCQ, Social Communication Questionnaire; SRS, Social Responsiveness Scale.

^a^ Reflects the standardized mean difference between indicator score distributions of the non‐ASD/DD and ASD groups, indexed by a Cohen's *d* threshold of ≥1.25.

^b^ Reflects within‐group (nuisance) correlations among indicators, indexed by a Pearson's *r* threshold of <.30.

**FIGURE 2:**
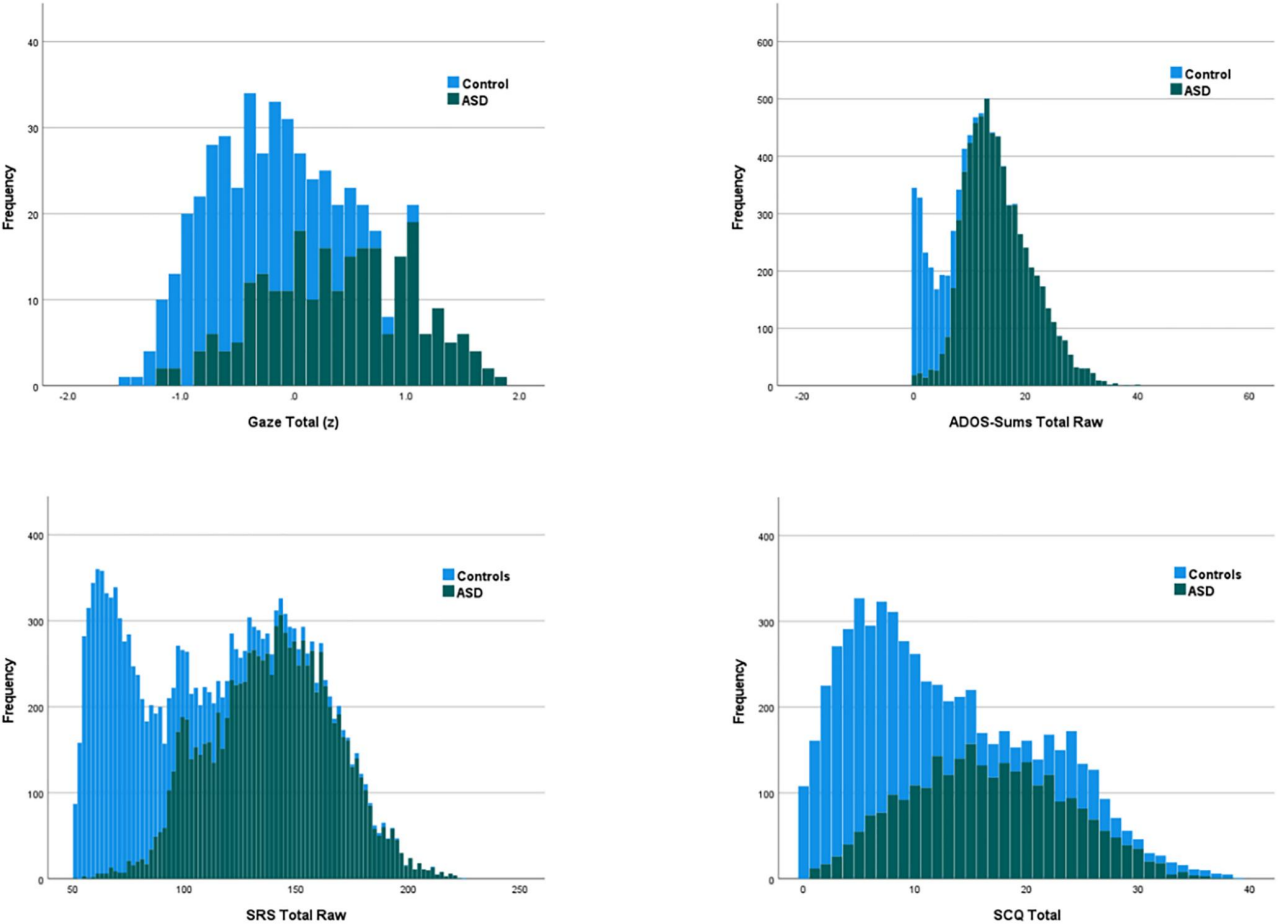
Stacked frequency distributions for ASD (green) and control (light blue) groups across total gaze and autism symptom measures, separately by indicator set. ASD, autism spectrum disorder.

Score distributions were highly variable; some showed relatively normal distribution (Gaze), others significant positive skew (SCQ), and some bimodal distributions (ADOS, SRS). Score distributions are not strong indicators of latent distributions (Ruscio et al., 2006), but the presence of different types of observed distributions ensures that the full pattern of results is not driven by peculiarities of the observed scores (see Figure 2).

### Latent structure

**FIGURE 3:**
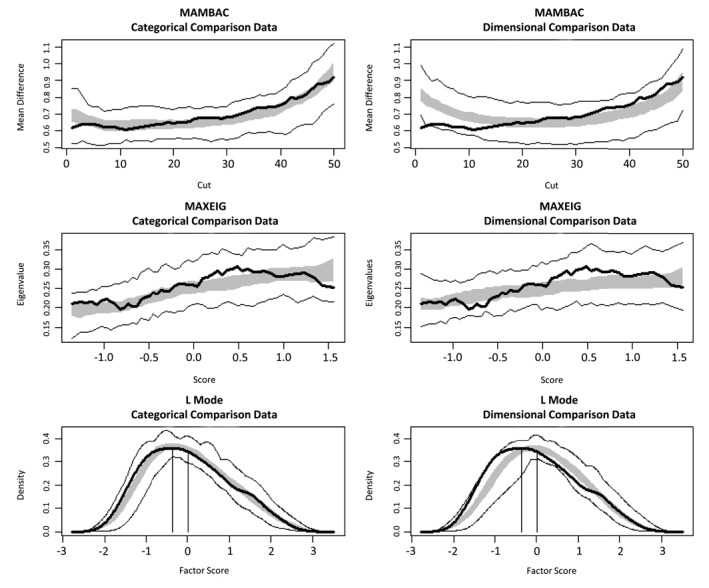
Taxometric analyses of Gaze‐7‐dx indicators. Each row displays results for one taxometric method (MAMBAC, MAXEIG, and L‐Mode). Graph pairs show results for the empirical data (dark line) superimposed on results for comparison datasets simulated using either categorical (left) or dimensional (right) structure; lighter lines represent minimum and maximum simulated values at each data point, and the gray area denotes the middle 50% of simulated values. MAMBAC, mean above minus below a cut; MAXEIG, maximum eigenvalue.

The sample size for each taxometric procedure ranged from *N* = 512 to 12,705 (see Table 1). Mean CCFIs for all indicator sets fell above 0.50. Nine of 10 indicator sets had mean CCFIs in the range of strong support for categorical structure (≥0.55; Table 2 and Figures S1–S9), including the primary gaze indicator set, which showed clear differentiation across taxometric procedures (Figure 3). Only one individual CCFI value fell below 0.50: for Gaze‐7‐qt, which had the weakest average indicator validity of any set. The pattern of support for categorical structure was similar when raw gaze metrics were used as indicators (Figure S10) and when participants with reduced cognitive and language ability were excluded (Table S4), and slightly stronger when indicators with low validity (*d* < 0.80) were excluded (Table S5).

**TABLE 2:** CCFI and LCA 2‐class agreement across all indicator sets.

Indicator set	MAMBAC	MAXEIG	L‐mode	Mean	Tax estimated base rate	LCA2‐class base rate	ASD diagnosis base rate	LCA 2‐class agreement			
	CCFI	CCFI	CCFI	CCFI	%	%	%	Kappa	Overall %	Sens %	Spec %
Gaze‐7‐dx	0.684	0.559	0.640	0.627	43.4%	39.6%	47.9%	0.535	76.9%	67.3%	85.8%
Gaze‐3‐dx	0.595	0.513	0.582	0.563	50.8%	46.1%	47.9%	0.518	76.0%	73.1%	78.7%
Gaze‐7‐qt	0.548	0.510	0.496	0.518	45.9%	45.4%	47.9%	0.356	68.0%	62.9%	72.7%
Gaze‐3‐qt	0.592	0.513	0.568	0.558	52.3%	45.6%	47.9%	0.510	75.6%	72.7%	78.3%
ADOS‐Items	0.581	0.563	0.515	0.553	48.6%	71.7%	80.6%	0.624	86.2%	85.9%	87.2%
ADOS‐Sums	0.624	0.638	0.636	0.633	56.2%	63.2%	79.8%	0.589	82.7%	78.7%	98.2%
SRS‐original	0.780	0.659	0.704	0.714	54.1%	52.5%	60.4%	0.662	83.3%	79.6%	88.9%
SRS‐factors	0.832	0.697	0.706	0.745	84.3%	48.2%	60.4%	0.608	80.2%	90.4%	73.6%
SRS‐RDoC	0.772	0.817	0.645	0.745	37.4%	47.6%	60.4%	0.604	80.0%	91.0%	72.9%
SCQ	0.575	0.842	0.537	0.651	34.2%	47.7%	45.0%	0.466	73.4%	73.5%	73.4%

Abbreviations: ADOS, Autism Diagnostic Observation Schedule; CCFI, Comparative Curve Fit Index; LCA, latent class analysis; MAMBAC, mean above minus below a cut; MAXEIG, maximum eigenvalue; SCQ, Social Communication Questionnaire; SRS, Social Responsiveness Scale.

**FIGURE 4:**
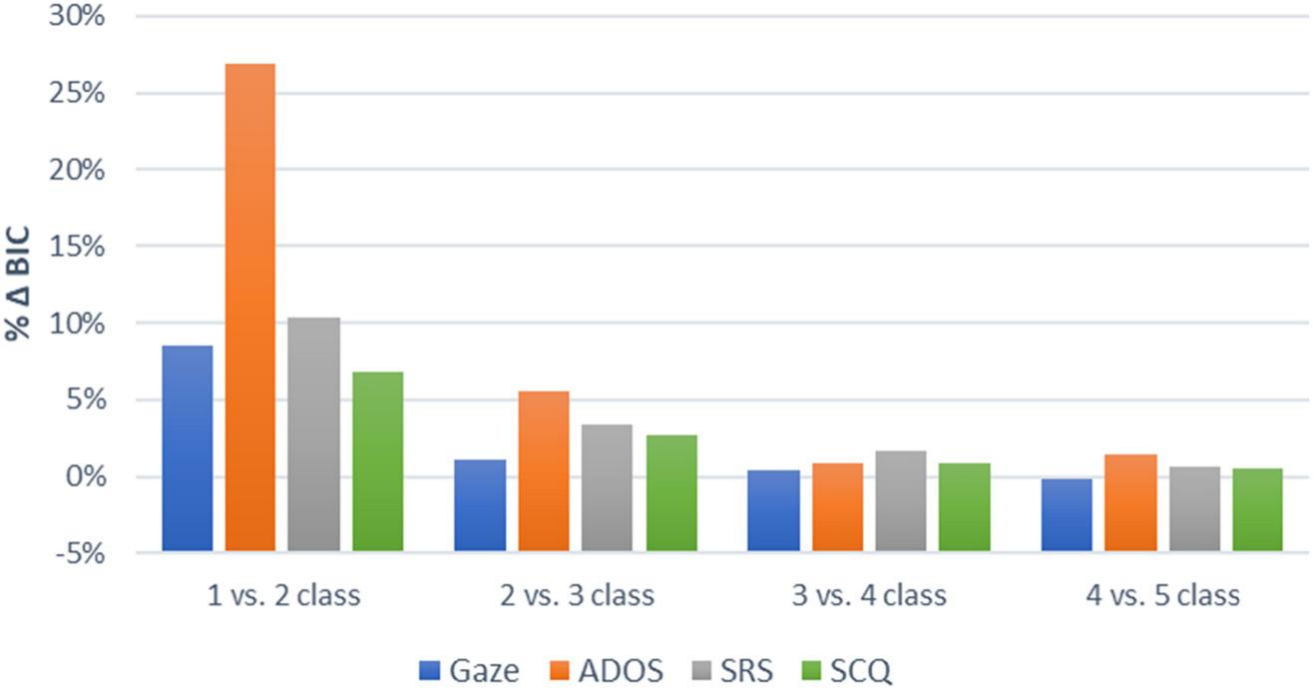
Improvement in model fit (% ΔBIC) across increasing classes for latent class analyses of each indicator type. Gaze is the Gaze‐7‐dx indicator set; ADOS is the ADOS‐Items indicator set; and SRS is the SRS‐Original indictor set. Highly similar results were obtained with other indicator sets for each measure. ADOS, Autism Diagnostic Observation Schedule; SCQ, Social Communication Questionnaire; SRS, Social Responsiveness Scale.

Results from LCA analyses supported the value of retaining a second latent class (Table S6), with stronger improvement in fit from 1 to 2 classes (4.9%–26.9% improvement in BIC) than subsequent class additions (Figure 4). Where additional classes improved fit, these classes tended to divide the control cases into classes resembling the NDD and non‐ASD/NDD groups or divide ASD cases based on symptom severity.

### Category base rate

For most indicator sets, the average taxometric (50.7%) and LCA 2‐class (50.8%) base rates were comparable, albeit slightly lower than, the ASD diagnosis (57.8%) base rate (Table 2). Under‐estimation may reflect imperfect sensitivity of most ASD measures, particularly to cognitively able presentations (Frazier et al., 2012). Absolute discrepancies across indicator sets were more variable for diagnosis‐taxometric base rate differences (−24% to +32%) but were closer for diagnosis‐LCA base rate differences (−3% to +17%).

### Latent class agreement with ASD diagnosis

LCA classifications showed adequate‐to‐very good agreement (*κ* = .356–.662; 68%–86%) with ASD diagnosis across most indicator sets (Table 2 and Figure S11). LCA classifications tended to have higher specificity than sensitivity for gaze and ADOS indicator sets, whereas most questionnaire‐based indicator sets showed greater sensitivity than specificity.

### Demographic and clinical features

Cases were organized by indicator set scores and classified into taxometric groupings that corresponded in size to the estimated base rate. Cases were classified into LCA groupings based on their posterior membership probabilities. Across all indictor sets, classifications and total scores were highly correlated with ASD diagnosis and quantitative measures; had small negative correlations with age, small positive correlations with male sex, small‐to‐moderate negative correlations with IQ, and moderate positive correlations with internalizing and externalizing problems, consistent with the substantial mental health comorbidity in ASD cases (Table 3). Interestingly, total scores showed stronger relationships with external correlates than classifications did.

**TABLE 3:** Concurrent (blue) and discriminant (purple) validity of categorical empirical classifications and continuous gaze and symptom measures with demographic and clinical measures.

	Gaze‐7‐dx		
	Taxometric classifications *r*	LCA classifications *r*	Total predicted gaze *r*
ASD diagnosis	0.46	0.54	0.55
ADOS severity	0.29	0.39	0.40
SRS total *T*‐score	0.35	0.35	0.40
IQ	−0.19	−0.25	−0.33
Language	−0.18	−0.24	−0.33
Internalizing problems	0.02	−0.04	0.01
Externalizing problems	0.08	0.06	0.05

	ADOS‐sums		
	Taxometric classifications *r*	LCA classifications *r*	Total raw score *r*
ASD diagnosis	0.56	0.64	0.66
Age	−0.12	−0.12	−0.15
Sex (male)	0.16	0.16	0.17
IQ	−0.29	−0.23	−0.43
Internalizing problems	−0.04	−0.01	−0.09
Externalizing problems	−0.01	0.01	−0.02

	SRS‐original		
	Taxometric classifications *r*	LCA classifications *r*	Total *T*‐score *r*
ASD diagnosis	0.68	0.67	0.73
Age	−0.02	−0.02	−0.01
Sex (male)	0.21	0.21	0.20
IQ	−0.24	−0.25	−0.32
Internalizing problems	0.44	0.43	0.53
Externalizing problems	0.34	0.35	0.44

	SCQ		
	Taxometric classifications *r*	LCA classifications *r*	Total raw score *r*
ASD diagnosis	0.34	0.47	0.40
Age	−0.22	−0.24	−0.20
Sex (male)	0.14	0.17	0.17
IQ	−0.38	−0.37	−0.51
Internalizing problems	0.07	0.13	0.12
Externalizing problems	0.05	0.10	0.11

Abbreviations: ADOS, Autism Diagnostic Observation Schedule; ASD, autism spectrum disorder; LCA, latent class analysis; SCQ, Social Communication Questionnaire; SRS, Social Responsiveness Scale.

## DISCUSSION

This examination, the largest and most comprehensive to date, indicates that ASD might be among a small number of psychopathology conditions with categorical structure (Haslam et al., 2020). Importantly, identification of categorical structure using objective gaze indicators demonstrates that these results are not simply a function of shared method variance (Podsakoff et al., 2003), rater biases or expectations (Beauchaine & Waters, 2003; McGrath et al., 2009) and that this structure is reflected in a key cognitive phenotype of ASD and neurodevelopment—social attention (Constantino et al., 2017; Frazier et al., 2017). Additional studies with other biomarkers showing good differentiation of ASD and non‐ASD phenotypes are warranted. Extending analyses to other cognitive, physiological, and neural systems measures, such as automated facial expression analysis (Trevisan et al., 2018) and pupillometry (de Vries et al., 2021), will be key for improving precision of ASD classification and assessment.

Latent categorical structure has important implications for nosology and assessment. First, the present results support the broad DSM‐5 conceptualization, with co‐occurrence of SCI and RRB symptoms and a wide range of severity within the ASD category. Additional research is needed to identify whether the specific criteria and exemplars listed in DSM‐5 optimally identify the ASD category. This will be key for the revision of future diagnostic systems, as will the development and refinement of symptom measures. While existing measures show good accuracy (Kim & Lord, 2012), the present results suggest that, rather than simply measuring degrees of symptom severity, measures should also provide estimates of the post‐test probability of a categorical ASD diagnosis. These values can be used in an evidence‐based medicine fashion to enhance clinical judgment. In this framework, post‐test probabilities can inform whether additional evaluation might be needed, when less intensive or non‐specific interventions may be warranted, or—when the probability is sufficiently high—more ASD‐specific or intensive interventions should be initiated (Frazier, Coury, et al., 2021).

Current findings have several implications for research design and analysis. For example, group designs need to sample the full range of cases within the ASD category, while quantitative trait designs need to consider the underlying latent distributions and how these might influence findings. Despite the identification of categorical structure, the use of quantitative scores is still important as these scores often show stronger correlations with other measures. Further investigations into the BAP in first‐degree relatives are needed. While present findings suggest that these traits are sub‐threshold, it is unclear whether BAP might itself represent a discrete behavior pattern or the end of a neurotypical continuum.

Primary limitations include the availability of a single ASD biomarker sample with sufficient indicator validity and the inclusion of indicator sets with less‐than‐optimal characteristics. The combination of cross‐cultural cohorts is unlikely to induce categorical structure because the latent classifications were consistent with ASD in both cohorts and prior work with this dataset found no substantial cultural influences on social attention (Frazier, Uljarevic, et al., 2021). Moreover, sub‐optimal indicator validity and high nuisance correlations should have biased results *away* from detecting a latent category (Ruscio et al., 2010). Some datasets had smaller proportions of NDD cases, which could have biased results toward the categorical structure. However, indicator sets with a higher proportion of NDD cases were present to offset this possibility. Confound may further be introduced by admixing samples drawn from separate populations who might differ on numerous characteristics other than the target construct. Yet, the categorical structure was supported across indicator sets with diverse sample compositions, including in single‐sample data and when participants with reduced cognitive and language ability were excluded. In addition, it is important to acknowledge the presence of multiple family members in the combined dataset. This may impact MAXEIG but should not influence the results of other taxometric procedures and was explicitly accounted for, where possible, in LCA. Finally, taxometric methods are not the only procedures for evaluating between categorical and dimensional models (Borsboom et al., 2016). Nevertheless, simulations have demonstrated that the CCFI utilized here is accurate at distinguishing dimensional and categorical structure in >99% of cases, under a wide range of conditions (Ruscio et al., 2018). Lastly, whereas taxometric procedures can detect only a single boundary (between two groups) at a time, this does not rule out the existence of additional groupings. Testing for further boundaries or subtypes *within* the ASD grouping is a potential avenue for additional investigation.

## CONCLUSION

ASD appears to be a qualitatively distinct category at the levels of behavioral symptoms and social attention, but additional replication is warranted. These findings support the broad structure of DSM‐5 ASD diagnosis and next generation diagnostic systems should maintain the ASD category. Future studies may consider this structure in design and analytic methods. Clinical investigations are needed to identify the optimal symptom measurements and evidence‐based assessment procedures for ASD identification and outcome tracking.

## AUTHOR CONTRIBUTIONS

**Thomas W. Frazier**: Conceptualization, Data curation, Formal analysis, Investigation, Methodology, Project administration, Writing – original draft, Writing – review & editing. **Lacey Chetcuti**: Validation, Writing – original draft, Writing – review & editing. **Fouad A. Al‐Shaban:** Data curation, Validation, Writing – review & editing. **Nick Haslam**: Methodology, Writing – review & editing. **Iman Ghazal**: Data curation, Project administration, Writing – review & editing. **Eric W. Klingemier:** Data curation, Project administration, Writing – original draft. **Mohammed Aldosari**: Data curation, Project administration, Writing – review & editing. **Andrew J. O. Whitehouse**: Validation, Writing – review & editing. **Eric A. Youngstrom**: Validation, Writing – review & editing. **Antonio Y. Hardan**: Validation, Writing – review & editing. **Mirko Uljarević**: Conceptualization, Data curation, Formal analysis, Investigation, Methodology, Project administration, Writing – original draft, Writing – review & editing.

## CONFLICT OF INTEREST STATEMENT

T.W.F. has received funding or research support from, acted as a consultant to, received travel support from, and/or received a speaker's honorarium from Quadrant Biosciences, Impel NeuroPharma, F. Hoffmann‐La Roche AG Pharmaceuticals, the Cole Family Research Fund, Simons Foundation, Ingalls Foundation, Forest Laboratories, Ecoeos, IntegraGen, Kugona LLC, Shire Development, Bristol‐Myers Squibb, Roche Pharma, National Institutes of Health, and the Brain and Behavior Research Foundation and has an investor stake in Autism EYES LLC. EAY has consulted with Lundbeck, Supernus, Pearson, and Western Psychological Services about psychological assessment, and received royalties from Guilford Press and the American Psychological Association. He is co‐founder and president of Helping Give Away Psychological Science (HGAPS.org). The remaining authors have declared that they have no competing or potential conflicts of interest.

## ETHICAL CONSIDERATIONS

Ethics approval was not required as data were publicly available and de‐identified.

## Supporting information

Supporting Information S1

## Data Availability

The data that support the findings of this study are available from the corresponding author upon reasonable request.
