# Supplementary material for: Categorical versus dimensional structure of autism spectrum disorder: A multi‐method investigation
Source: JCPP Adv. 2023 Feb 21;3(2):e12142. doi: 10.1002/jcv2.12142 (PMC10519739; doi:10.1002/jcv2.12142)
Supplement: Supplementary file 1 — Supporting Information S1 [file JCV2-3-e12142-s001.docx]

**Supporting Information**

**Table S1**. Additional sample characteristics by measure/indicator set.

| **Measure** | **Samples** | | **Age**  **(Mean)** | | | **% Male** | | | **IQ**  **(Mean SS)** | | | **Internalizing**  **(Mean T-Score)** | | | **Externalizing**  **(Mean T-Score)** | | |
| --- | --- | --- | --- | --- | --- | --- | --- | --- | --- | --- | --- | --- | --- | --- | --- | --- | --- |
|  |  | **N** | **NT** | **NDD** | **ASD** | **NT** | **NDD** | **ASD** | **NT** | **NDD** | **ASD** | **NT** | **NDD** | **ASD** | **NT** | **NDD** | **ASD** |
| Gaze | Autism EYES | 512 | 6.6 | 6.4 | 7.6 | 59% | 76% | 79% | - | 88 | 75 | 46 | 64 | 60 | 44 | 64 | 57 |
| ADOS-Items | SSC/AGRE/NDAR | 2851/1342/8512 | 5.8 | 13.6 | 9.6 | 61% | 69% | 82% | 103 | 111 | 86 | 46 | 50 | 60 | 46 | 51 | 57 |
| ADOS-Sums | SSC/AGRE/NDAR | 2321/296/5527 | 10.0 | 16.5 | 11.5 | 62% | 69% | 84% | 103 | 111 | 93 | 50 | 53 | 61 | 47 | 52 | 57 |
| SRS | SSC/AGRE/NDAR/  HBN*/Norms | 5116/3399/5169/  1889/1182 | 9.7 | 10.5 | 9.8 | 59% | 63% | 79% | 109 | 98 | 88 | 48 | 58 | 60 | 46 | 57 | 56 |
| SCQ | SSC/NDAR/HBN | 2179/1896/1965 | 6.8 | 8.8 | 7.8 | 51% | 63% | 83% | 106 | 98 | 82 | 48 | 56 | 60 | 47 | 55 | 56 |

*Note.* ADOS=Autism Diagnostic Observation Schedule; SRS=Social Responsiveness Scale; SCQ=Social Communication Questionnaire; RBS-R=Repetitive Behavior Scale-Revised. Autism EYES=Cleveland Clinic and Qatar Biomedical Research Institute cross-cultural cohort, SSC=Simons Simplex Collection, AGRE=Autism Genetic Resource Exchange, NDAR=National Database for Autism Research, HBN=Healthy Brain Network, Norms=SRS-2 normative data, IQ=full scale intelligence quotient presented in standard score units (M=100, SD=15). Internalizing and externalizing scores presented in T-score units (M=50, SD=10).

**Table S2**. ASD diagnostic procedures by database.

| **Database** | **Diagnostic procedures** |
| --- | --- |
| Autism EYES | Best estimate clinical or research diagnoses informed by the ADOS. |
| AGRE | Best estimate clinical or research diagnoses informed by the ADOS and ADI-R. |
| HBN | Best estimate clinical or research diagnoses informed by KSADS-COMP, ADOS, and ADI-R. |
| NDAR | Informant-reported or best estimate clinical or research diagnoses informed by instruments including the ADI-R, SRS, and SCQ. |
| SRS Norm | Diagnoses based on mixture of gold-standard assessment instruments and informant-reported clinical diagnoses |
| SSC | Best estimate clinical or research diagnoses informed by the ADOS and ADI-R. |

*Note.* AGRE=Autism Genetic Resource Exchange, Autism EYES=Cleveland Clinic and Qatar Biomedical Research Institute cross-cultural cohort, HBN=Healthy Brain Network, Norms=SRS-2 normative data, NDAR=National Database for Autism Research, SSC=Simons Simplex Collection, ADOS = Autism Diagnostic Observation Schedule, ADI-R = Autism Diagnostic Interview-Revised, KSADS-COMP = Schedule for Affective Disorders and Schizophrenia-Children’s version, SRS = Social Responsiveness Scale, SCQ = Social Communication Questionnaire.

**Table S3.** ADOS items comprising indicator sets.

| **Indicator set** | **ADOS items** |
| --- | --- |
| ADOS-Items | Unusual Eye Contact; Shared Enjoyment in Interaction; Response to Joint Attention; Imagination/Creativity; Quality of Social Overtures; Gestures; Unusual Sensory Interest in Play Material/Person; Hand and Finger and Other Complex Mannerisms |
| ADOS-Sums | (i) Gestures; Unusual Eye Contact; Response to Joint Attention; (ii) Overall Level of Non-Echoed Spoken Language; Conversation; Shared Enjoyment in Interaction; Quality of Social Overtures; Quality of Social Response; Amount of Reciprocal Social Interaction; Overall Quality of Rapport; Imagination/Creativity; (iii) Speech Abnormalities Associated with Autism; Immediate Echolalia; Stereotyped/Idiosyncratic Use of Words or Phrases; Unusual Sensory Interest in Play Material/Person; Hand and Finger and Other Complex Mannerisms |

*Note.* ADOS = ADOS = Autism Diagnostic Observation Schedule.

**Supplemental Methods.**

Sampling frameworks of utilized datasets

The Qatar Foundation and US-Cleveland Clinic (Autism EYES) cohort consists of US and Qatari youth (aged 1-17) referred to multi-disciplinary ASD evaluation clinics and neurotypical controls or controls with other developmental disabilities (DD) recruited from local primary care clinics, unaffected siblings of clinic patients, or from researcher contacts. ASD diagnosis was based consensus and informed by a parent interview and psychosocial history conducted by a psychologist, medical evaluation and developmental history confirmed by a physician, and the Autism Diagnostic Observation Schedule-Second Edition (ADOS-2) completed by a reliable administrator. The ADOS-2 was administered to all referred cases in the US sample but only to confirm clinical ASD diagnosis or rule out possible ASD in the Qatar sample. Inclusion in the neurotypical control group required no evidence of any developmental disability or neuropsychiatric condition as reported by parents/caregivers. Inclusion in the DD group required any other neurodevelopmental or neuropsychiatric disorder diagnosis besides ASD.

The Autism Genetic Research Exchange (AGRE; [www.agre.org](http://www.agre.org)) is an open-access repository of clinical and genetic information on individuals with multiplex ASD. Recruitment into AGRE was primarily through community events and social media, with priority was given to families with two or more immediate family members with ASD. Inclusion criteria includes a diagnosis of ASD (including autistic disorder, pervasive developmental disorder not otherwise specified, and Asperger’s disorder) and having an English-speaking parent. Diagnostic procedures included the ADOS and ADI-R.

The Healthy Brain Network (HBN; <https://healthybrainnetwork.org/>) cohort comprises children and adolescents (aged 5-21) referred from the community based on having psychiatric symptoms of concern. Inclusion criteria included age 5-21 years, capacity to understand the study and provide informed consent/assent, and English fluency. Exclusion criteria included serious neurological (specific or focal) disorders preventing full participation in the protocol, acute encephalopathy (brain dysfunction) caused by an injury to the brain or disease, known neurodegenerative disorders, recent diagnosis (within the past 6 months) of Schizophrenia, Schizoaffective Disorder, or Bipolar Disorder, manic or psychotic episode within the past 6 months without current ongoing treatment, new onset (within the last 3 months) of untreated suicidality or homicidality, history of lifetime substance dependence requiring chemical replacement therapy, and acute intoxication at time of any study visit. All participants were administered a computerized web-based version of the Schedule for Affective Disorders and Schizophrenia-Children’s version (KSADS) and those suspected of ASD were administered the ADOS and ADI-R.

The National Database for Autism Research (NDAR) is a research data repository that includes datasets from hundreds of different research laboratories and projects involving distinct sampling frames and inclusion/exclusion criteria. Among the diagnostic procedures are the ADI-R, ADOS, SRS, and SCQ. Further information is available at <https://nda.nih.gov/>.

The Social Responsiveness Scale (SRS) norming sample encompasses typically developing individuals without any clinical diagnosis.

The Simons Simplex Collection (SSC; <https://www.sfari.org/>) cohort comprises individuals with simplex ASD. Recruitment into SSC was through partnerships with local service providers and parent and advocacy groups, and web postings and radio and television ads. Inclusion criteria included age 4-21 years. Exclusion criteria included presence of conditions that might compromise validity of diagnostic instruments such as nonverbal mental age below 18 months, severe neurological deficits, birth trauma, perinatal complications, or genetic evidence of Fragile X or Down syndromes. Diagnostic procedures included the ADOS and ADI-R.

Latent class analysis (LCA) model estimation and fit evaluation

The following statistical indices assessed LCA model fit: the Akaïke Information Criterion (AIC; Akaike, 1987) the Bayesian Information Criterion (BIC; Raftery, 1995) and the sample-size adjusted BIC (saBIC), with lower values indicating better fit. Preference was given to the BIC, as this index penalizes more heavily for model complexity (Nylund, Asparouhov, & Muthén, 2007). Entropy was also included to evaluate classification accuracy. Information criteria used to evaluate the fit of LCA models, particularly those implemented in large samples, may not reach a local minimum (Morin, 2016). The magnitude of improvement in model fit across increasing classes (1 vs. 2, 2 vs. 3, etc.) also provides information about the importance of subsequent distinctions beyond a 2-class structure (Raftery, 1995). In these situations, it can be useful to examine relative improvement in fit (ΔBIC and % change in BIC across successive models) to determine whether retaining additional classes (3+) is warranted to more accurately describe the data (Morin, 2016; Morin et al., 2011; Petras & Masyn, 2010). Additionally, LCA models may be less accurate than taxometric procedures at distinguishing dimensional versus 2-class categorical structure, but more accurate in specifying the full model when additional classes are present (Cleland, Rothschild, & Haslam, 2000; McGrath, 2008; McGrath & Walters, 2012; Walters, McGrath, & Knight, 2010). For this reason, LCA models specifying one-through-five classes were examined to determine whether these additional classes identify unique patterns not related to ASD/control status.

**Figure S1**. Taxometric analyses of Gaze-3-dx indicator set.


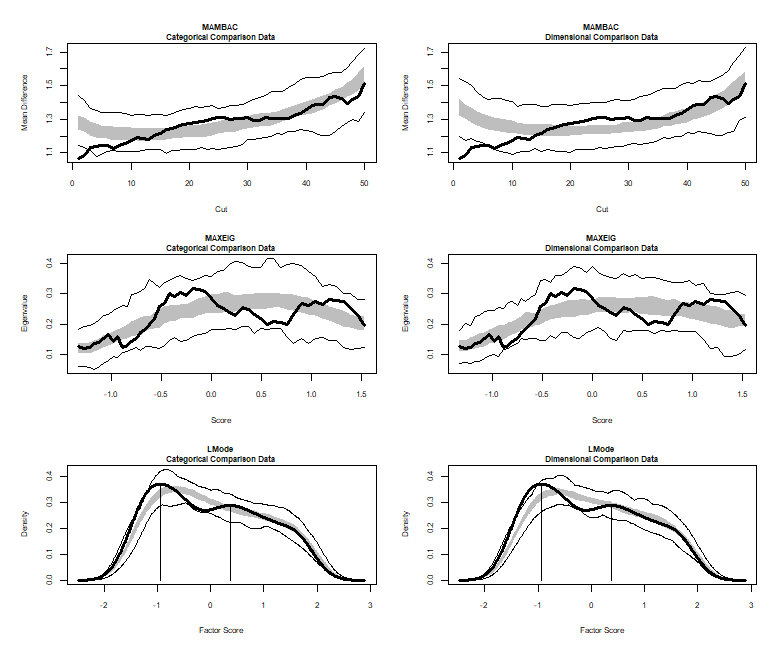


**Figure S2**. Taxometric graphs for the Gaze-7-qt indicator set.


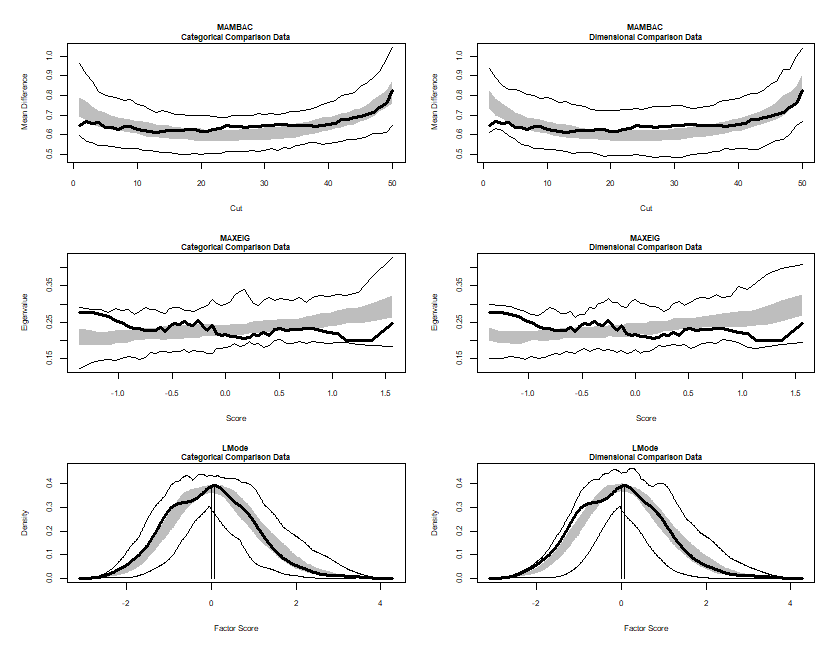


**Figure S3**. Taxometric graphs for the Gaze-3-qt indicator set.


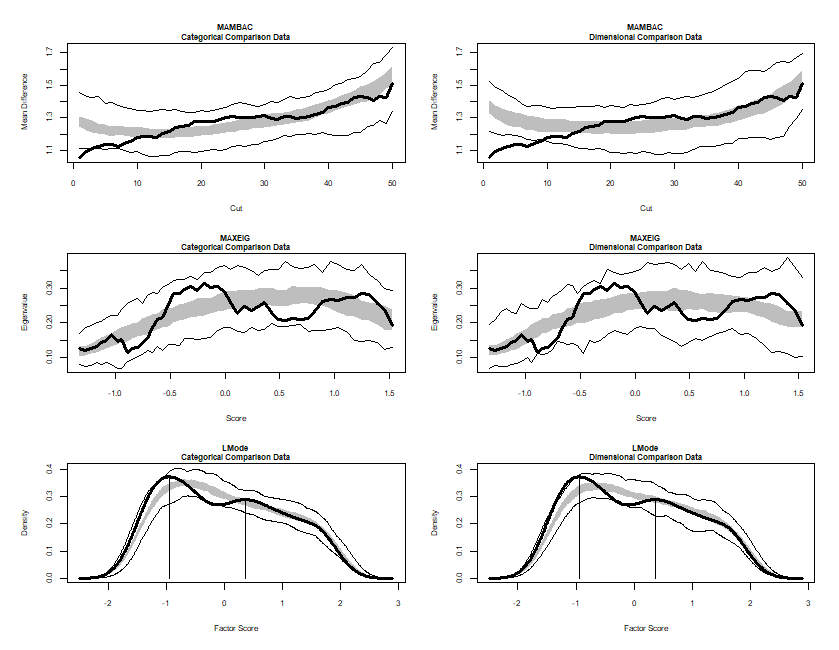


**Figure S4**. Taxometric analyses of ADOS-Items indicator set.


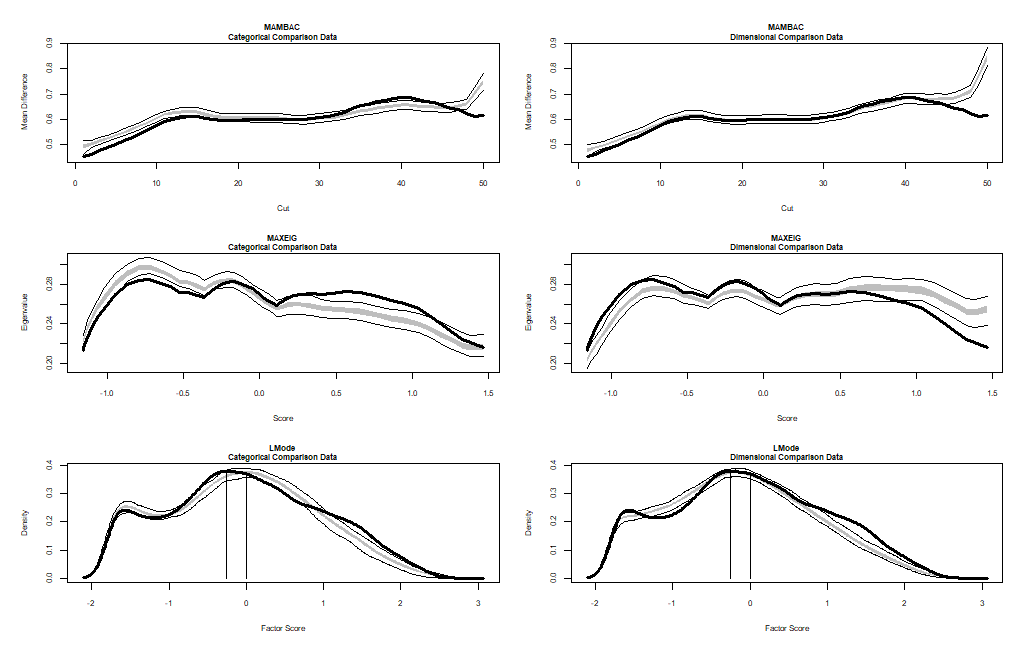


**Figure S5**. Taxometric graphs for the ADOS-Sums indicator set.


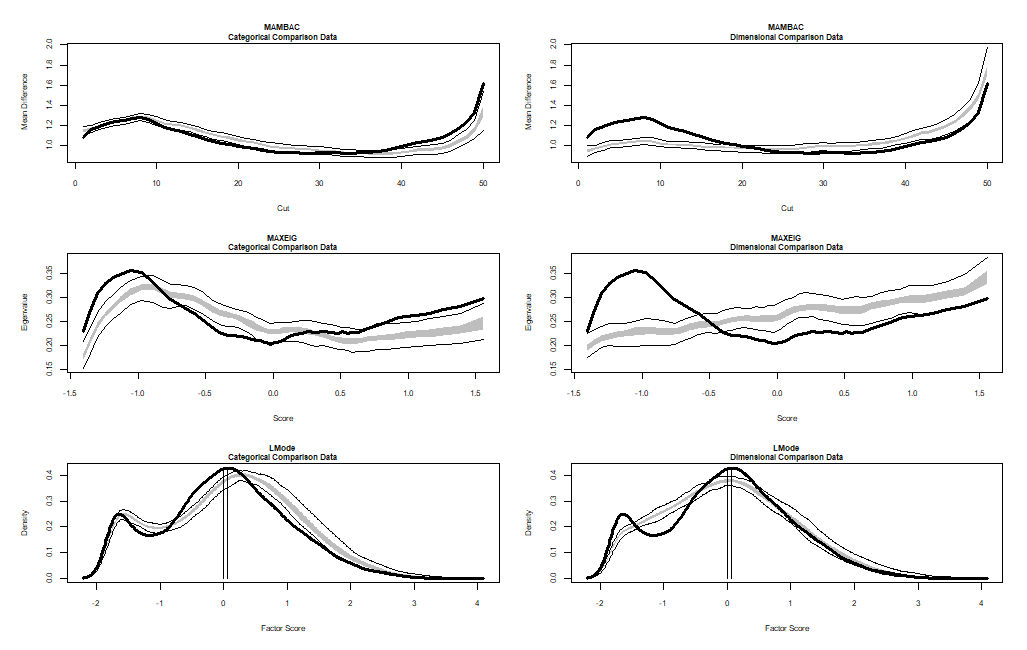


**Figure S6**. Taxometric analyses of SRS-Original indicator set.


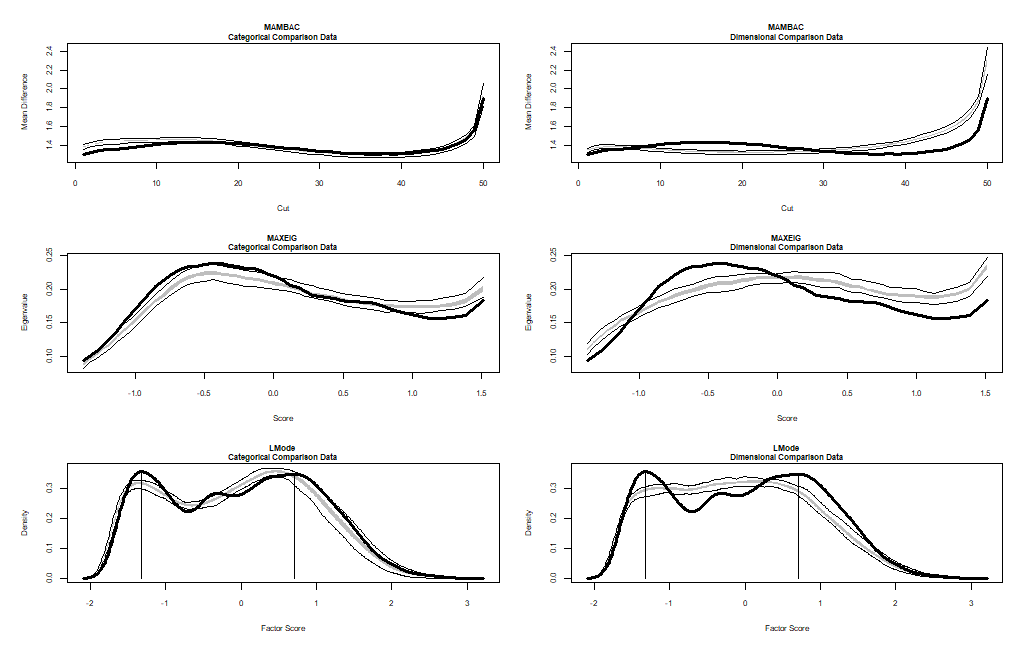


**Figure S7**. Taxometric analyses of SRS-Factors indicator set.


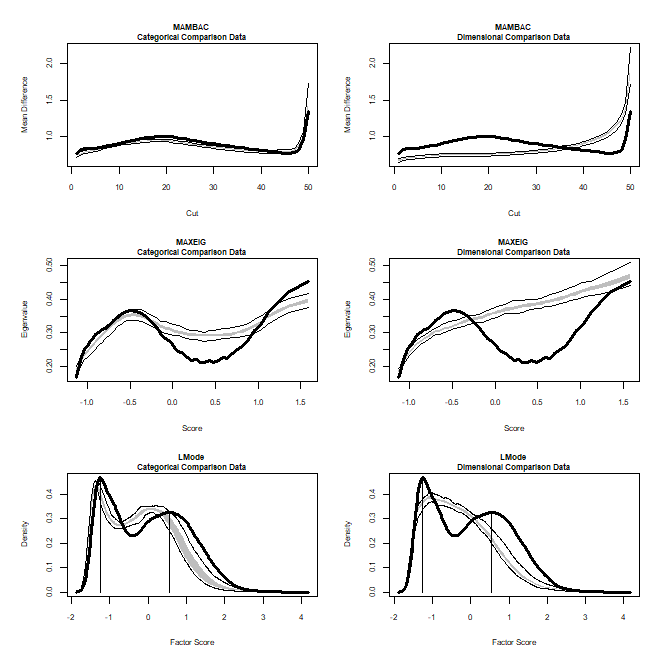


**Figure S8**. Taxometric analyses of SRS-RDoC indicator set.


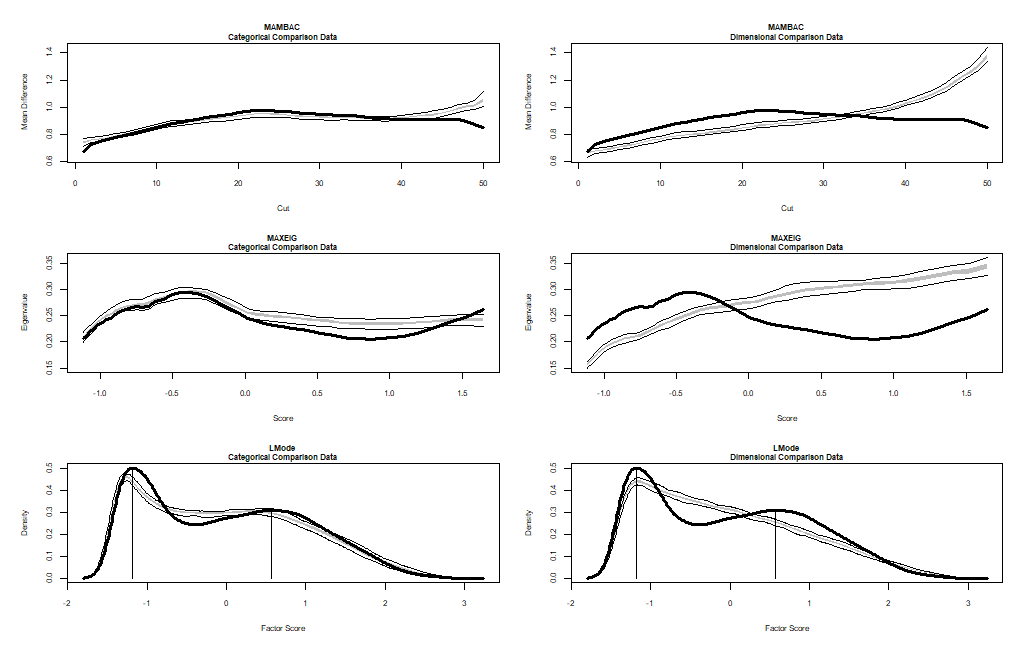


**Figure S9**. Taxometric analyses of SCQ indicator set.


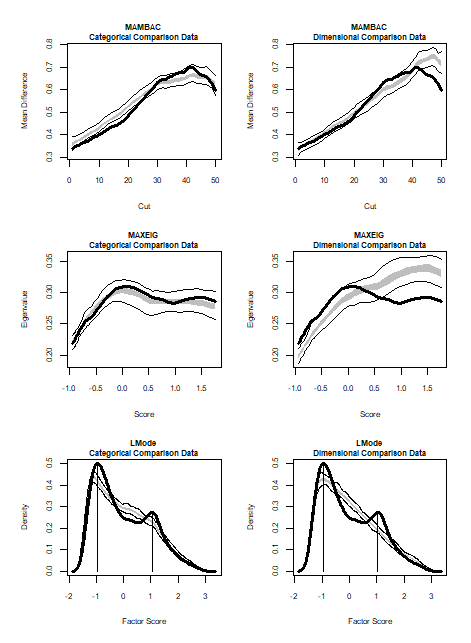


**Figure S10.** Taxometric analyses of gaze data in their raw form.


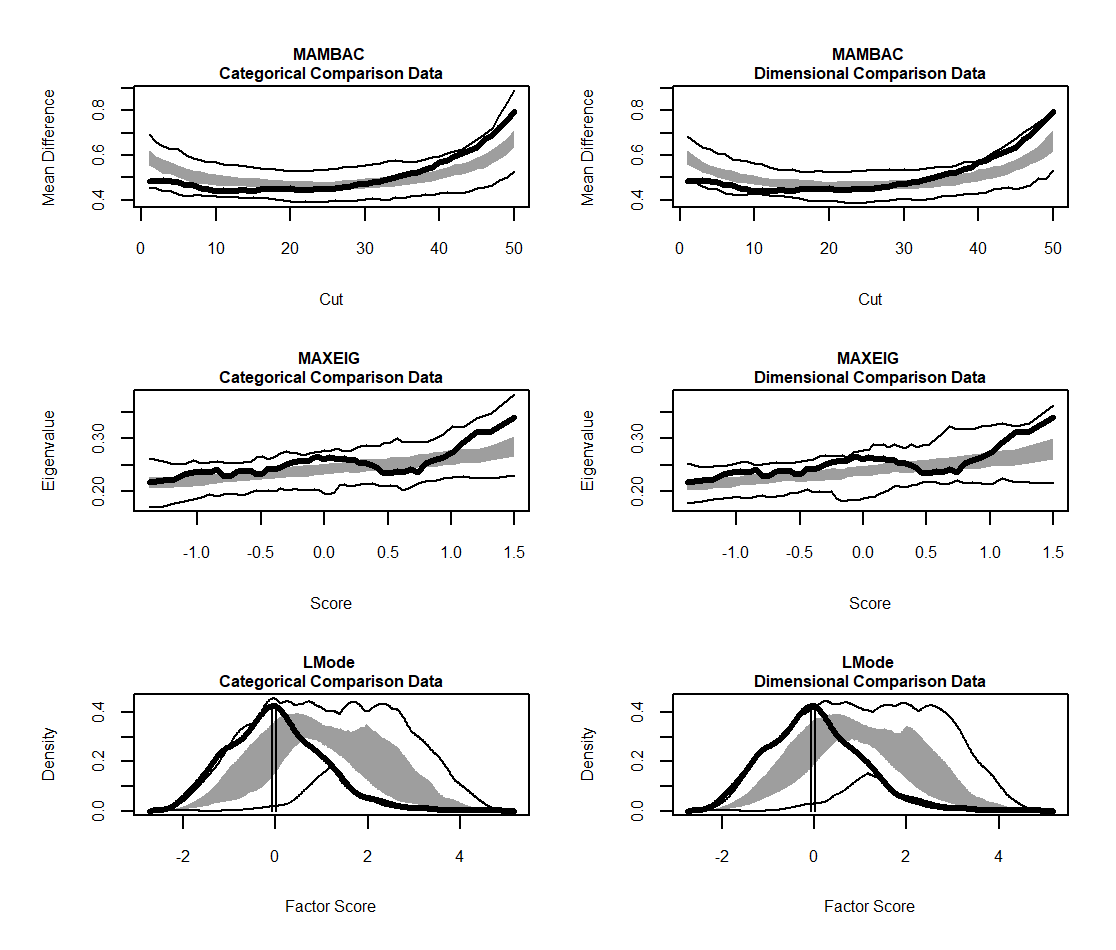


**Table S4**. Comparative Curve Fit Index (CCFI) results for selected gaze, ADOS, SRS, and SCQ indicators sets excluding participants with low cognitive or language scores (SS<70), intellectual disability, or global developmental delay.

| **Indicator Set** | **MAMBAC** | **MAXEIG** | **L-Mode** | **Mean** | **Tax Estimated**  **Base Rate** | **ASD Diagnosis**  **Base Rate** |
| --- | --- | --- | --- | --- | --- | --- |
|  | **CCFI** | **CCFI** | **CCFI** | **CCFI** | **%** | **%** |
| Gaze-7-dx | 0.723 | 0.525 | 0.536 | 0.594 | 43.6% | 37.7% |
| Gaze-3-dx | 0.616 | 0.488 | 0.572 | 0.559 | 44.8% | 37.7% |
| Gaze-7-qt | 0.535 | 0.477 | 0.517 | 0.510 | 47.1% | 37.7% |
| Gaze-3-qt | 0.601 | 0.483 | 0.554 | 0.546 | 44.8% | 37.7% |
| ADOS-Items | 0.594 | 0.493 | 0.750 | 0.613 | 51.6% | 74.0% |
| ADOS-Sums | 0.659 | 0.642 | 0.613 | 0.638 | 69.2% | 94.4% |
| SRS-Original | 0.700 | 0.703 | 0.704 | 0.703 | 43.3% | 36.8% |
| SRS-Factors | 0.855 | 0.657 | 0.771 | 0.761 | 62.2% | 36.8% |
| SRS-RDoC | 0.776 | 0.891 | 0.764 | 0.810 | 36.4% | 36.8% |
| SCQ | 0.462 | 0.740 | 0.551 | 0.584 | 35.0% | 31.3% |

*Note.* Samples sizes for indicator sets were: Gaze n=385, ADOS-Items n=3843, ADOS-Sums n=2696, SRS n=9802, and SCQ n=3625.

**Table S5**. Comparative Curve Fit Index (CCFI) results for selected gaze, ADOS, SRS, and SCQ indicators sets excluding indicators with low validity (d<.80).

| **Indicator Set** | **MAMBAC** | **MAXEIG** | **L-Mode** | **Mean** | **Revised**  **Validity** | **Tax Estimated**  **Base Rate** | **ASD Diagnosis**  **Base Rate** |
| --- | --- | --- | --- | --- | --- | --- | --- |
|  | **CCFI** | **CCFI** | **CCFI** | **CCFI** | **(d)** | **%** | **%** |
| Gaze-7-dx | 0.724 | 0.642 | 0.679 | 0.682 | 0.99 | 46.6% | 47.9% |
| ADOS-Items | 0.619 | 0.655 | 0.688 | 0.654 | 1.40 | 47.7% | 80.6% |
| SRS-Factors | 0.839 | 0.631 | 0.758 | 0.743 | 1.45 | 59.3% | 60.4% |
| SRS-RDoC | 0.772 | 0.817 | 0.645 | 0.745 | 1.42 | 36.4% | 60.4% |
| SCQ | 0.797 | 0.797 | 0.695 | 0.763 | 0.93 | 40.2% | 45.0% |

*Note.* Other gaze indicator sets were not included because they either had only 3 indicators with all indicators d>.80 (gaze-3-dx and gaze-3-qt) or because only one indicator was d>.80 (gaze-7-qt). ADOS-Sums and SRS-Original had all indicators d>.1.25.

**Table S6**. LCA model results for 1-5 class solutions, separately for each indicator set.

| **Gaze-7-dx** | | | | | | | | |
| --- | --- | --- | --- | --- | --- | --- | --- | --- |
| Model | par | LL | AIC | BIC | saBIC | entropy | ΔBIC | % ΔBIC |
| 1-class | 14 | -4705 | 9437 | 9497 | 9452 | - | - | - |
| 2-class | 22 | -4276 | 8596 | 8689 | 8620 | 0.841 | 808 | 8.5% |
| 3-class | 30 | -4199 | 8459 | 8586 | 8491 | 0.771 | 103 | 1.2% |
| 4-class | 38 | -4154 | 8384 | 8545 | 8424 | 0.796 | 41 | 0.5% |
| 5-class | 46 | -4134 | 8360 | 8555 | 8409 | 0.772 | -10 | -0.1% |
| **Gaze-3-dx** | | | | | | | | |
| Model | par | LL | AIC | BIC | saBIC | entropy | ΔBIC | % ΔBIC |
| 1-class | 6 | -2175 | 4362 | 4387 | 4368 | - | - | - |
| 2-class | 10 | -1750 | 3521 | 3563 | 3532 | 0.885 | 824 | 18.8% |
| 3-class | 14 | -1634 | 3296 | 3355 | 3311 | 0.876 | 208 | 5.8% |
| 4-class | 18 | -1609 | 3255 | 3331 | 3274 | 0.807 | 24 | 0.7% |
| 5-class | 22 | -1585 | 3213 | 3307 | 3237 | 0.815 | 24 | 0.7% |
| **Gaze-7-qt** | | | | | | | | |
| Model | par | LL | AIC | BIC | saBIC | entropy | ΔBIC | % ΔBIC |
| 1-class | 14 | -4947 | 9922 | 9981 | 9937 | - | - | - |
| 2-class | 22 | -4613 | 9270 | 9363 | 9293 | 0.750 | 618 | 6.2% |
| 3-class | 30 | -4503 | 9067 | 9194 | 9099 | 0.812 | 169 | 1.8% |
| 4-class | 38 | -4477 | 9030 | 9191 | 9070 | 0.718 | 3 | 0.0% |
| 5-class | 46 | -4449 | 8990 | 9185 | 9039 | 0.746 | 6 | 0.1% |
| **Gaze-3-qt** | | | | | | | | |
| Model | par | LL | AIC | BIC | saBIC | entropy | ΔBIC | % ΔBIC |
| 1-class | 6 | -2168 | 4349 | 4374 | 4355 | - | - | - |
| 2-class | 10 | -1745 | 3509 | 3552 | 3520 | 0.884 | 823 | 18.8% |
| 3-class | 14 | -1626 | 3279 | 3339 | 3294 | 0.878 | 213 | 6.0% |
| 4-class | 18 | -1602 | 3239 | 3316 | 3259 | 0.815 | 23 | 0.7% |
| 5-class | 22 | -1576 | 3197 | 3290 | 3220 | 0.819 | 26 | 0.8% |
| **ADOS-Items** | | | | | | | | |
| Model | par | LL | AIC | BIC | saBIC | entropy | ΔBIC | % ΔBIC |
| 1-class | 16 | -125619 | 251271 | 251390 | 251339 | - | - | - |
| 2-class | 25 | -91754 | 183559 | 183745 | 183666 | 0.999 | 67645 | 26.9% |
| 3-class | 34 | -84806 | 169680 | 169933 | 169825 | 0.915 | 13812 | 7.5% |
| 4-class | 43 | -83681 | 167448 | 167769 | 167632 | 0.906 | 2165 | 1.3% |
| 5-class | 52 | -81816 | 163737 | 164124 | 163959 | 0.896 | 3645 | 2.2% |
| **ADOS-Sums** | | | | | | | | |
| Model | par | LL | AIC | BIC | saBIC | entropy | ΔBIC | % ΔBIC |
| 1-class | 6 | -58221 | 116453 | 116495.1 | 116476 | - | - | - |
| 2-class | 10 | -55115 | 110250.2 | 110320.2 | 110288.5 | 0.754 | 6175 | 5.3% |
| 3-class | 14 | -53503 | 107034.2 | 107132.2 | 107087.7 | 0.810 | 3188 | 2.9% |
| 4-class | 18 | -53259 | 106554.3 | 106680.4 | 106623.2 | 0.785 | 452 | 0.4% |
| 5-class | 22 | -52990 | 106023.9 | 106178 | 106108.1 | 0.764 | 502 | 0.5% |
| **SRS-Original** | | | | | | | | |
| Model | par | LL | AIC | BIC | saBIC | entropy | ΔBIC | % ΔBIC |
| 1-class | 10 | -291453 | 582926 | 583003 | 582972 | - | - | - |
| 2-class | 16 | -261403 | 522838 | 522962 | 522911 | 0.938 | 60041 | 10.3% |
| 3-class | 22 | -251473 | 502989 | 503159 | 503089 | 0.916 | 19803 | 3.8% |
| 4-class | 28 | -246524 | 493105 | 493321 | 493232 | 0.903 | 9838 | 2.0% |
| 5-class | 34 | -244514 | 489096 | 489359 | 489251 | 0.887 | 3962 | 0.8% |
| **SRS-Factors** | | | | | | | | |
| Model | par | LL | AIC | BIC | saBIC | entropy | ΔBIC | % ΔBIC |
| 1-class | 10 | -310522 | 621065 | 621142 | 621110 | - | - | - |
| 2-class | 16 | -295401 | 590835 | 590958 | 590908 | 0.880 | 30184 | 4.9% |
| 3-class | 22 | -281602 | 563247 | 563417 | 563347 | 0.948 | 27541 | 4.7% |
| 4-class | 28 | -276413 | 552883 | 553099 | 553010 | 0.917 | 10318 | 1.8% |
| 5-class | 34 | -271040 | 542148 | 542411 | 542303 | 0.933 | 10688 | 1.9% |
| **SRS-RDoC** | | | | | | | | |
| Model | par | LL | AIC | BIC | saBIC | entropy | ΔBIC | % ΔBIC |
| 1-class | 14 | -307411 | 614850 | 614959 | 614914 | - | - | - |
| 2-class | 22 | -279736 | 559517 | 559687 | 559617 | 0.922 | 55272 | 9.0% |
| 3-class | 30 | -272978 | 546017 | 546249 | 546153 | 0.889 | 13438 | 2.4% |
| 4-class | 38 | -271017 | 542110 | 542403 | 542283 | 0.861 | 3845 | 0.7% |
| 5-class | 46 | -268673 | 537438 | 537794 | 537648 | 0.877 | 4609 | 0.8% |
| **SCQ** | | | | | | | | |
| Model | par | LL | AIC | BIC | saBIC | entropy | ΔBIC | % ΔBIC |
| 1-class | 27 | -53251 | 106556 | 106737 | 106651 | - | - | -- |
| 2-class | 55 | -49522 | 99155 | 99524 | 99349 | 0.820 | 7213 | 6.8% |
| 3-class | 83 | -47992 | 96149 | 96706 | 96442 | 0.838 | 2818 | 2.8% |
| 4-class | 111 | -47405 | 95032 | 95777 | 95424 | 0.823 | 929 | 1.0% |
| 5-class | 139 | -46996 | 94270 | 95203 | 94761 | 0.805 | 574 | 0.6% |

**Figure S11.** Venn diagram showing agreement between ASD diagnosis (labelled Dx ASD) and ASD classification based on LCA of the primary indicator set (Gaze-7-dx; labelled LCA ASD).


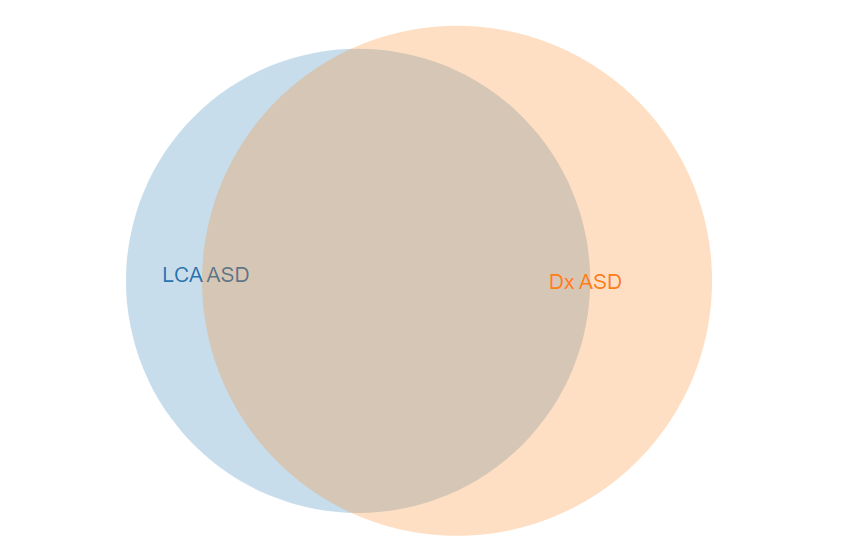


**References**

Akaike, H. (1987). Factor analysis and AIC. *Psychometrika, 52*, 317-332.

Cleland, C. M., Rothschild, L., & Haslam, N. (2000). Detecting latent taxa: Monte Carlo comparison of taxometric, mixture model, and clustering procedures. *Psychological Reports, 87*, 37-47.

McGrath, R. E. (2008). Inferential errors in taxometric analyses of ordered three-class constructs. *Journal of Personality Assessment, 90*(1), 11-25. doi:10.1080/00223890701356755

McGrath, R. E., & Walters, G. D. (2012). Taxometric Analysis as a General Strategy for Distinguishing Categorical From Dimensional Latent Structure. *Psychological Methods, 17*(2), 284-293. doi:10.1037/a0026973

Morin, A. J. (2016). Chapter 35: Person-centered research strategies in commitment research. In J. P. Meyer (Ed.), *Handbook of Employee Commitment* (pp. 490-508). Cheltenham, UK: Edward Elgar Publishing Limited.

Morin, A. J. S., Maïano, C., Nagengast, B., Marsh, H. W., Morizot, J., & Janosz, M. (2011). Growth mixture modeling of adolescents trajectories of anxiety: The impact of untested invariance assumptions on substantive interpretations. *Structural Equation Modeling, 18*, 613-648.

Nylund, K. L., Asparouhov, T., & Muthén, B. O. (2007). Deciding on the number of classes in latent class analysis and growth mixture modeling: A Monte Carlo simulation study. *Structural Equation Modeling, 14*(4), 535-569.

Petras, H., & Masyn, K. (2010). General growth mixture analysis with antecedents and consequences of change. . In D. W. A.R. Piquero (Ed.), *Handbook of Quantitative Criminology* (pp. 69-100). New York, NY: Springer.

Raftery, A. (1995). Bayesian model selection in social research. *Sociological Methodology, 25*, 111-163.

Walters, G. D., McGrath, R. E., & Knight, R. A. (2010). Taxometrics, polytomous constructs, and the comparison curve fit index: a Monte Carlo analysis. *Psychological Assessment, 22*(1), 149-156. doi:10.1037/a0017819
